# Supplementary material for: Content-rich biological network constructed by mining PubMed abstracts
Source: BMC Bioinformatics. 2004 Oct 8;5:147. doi: 10.1186/1471-2105-5-147 (PMC528731; doi:10.1186/1471-2105-5-147)
Supplement: Additional File 2 — The original results of the above study (non-essential files are deleted to keep the file size under the limit set by BMC bioinformatics). [file 1471-2105-5-147-S2.bz2 › chilibotAdditionalFile2/dip10/582ID9571026E10950/html/GHR_GH1.html]

 


 **GHR** and **GH1** 
  
Found 60 abstracts in PubMed, retrieved 10.  
 

 What does Google say? 
 PDF only 
| .edu only 

---

**Interactive relationship** (e.g. stimulation, inhibition, etc)

**Non-interactive relationship** (e.g. studied together, co-existance, homology, etc.)

- Pituitary growth hormone  [ **GH1** ]  GH is essential for postnatal growth in animal, it regulates numerous cellular functions by direct effect on it s receptor  **GHr**  in many different tissues.  Ref: 12096625 Yi Chuan Xue Bao, 2002
